# COVID-19 associated pulmonary aspergillosis (CAPA) in a pregnant woman

**DOI:** 10.4322/acr.2021.313

**Published:** 2021-08-20

**Authors:** Shruti Agrawal, Ravi Hari Phulware

**Affiliations:** 1 All India Institute of Medical Sciences, Department of Pathology & Laboratory Medicine, Rishikesh, Uttarakhand, India

**Keywords:** COVID-19, Pulmonary Aspergillosis, Respiratory Distress Syndrome,, Fungi, SARS-CoV-2

## Abstract

Viral or bacterial co-infections with severe acute respiratory syndrome coronavirus 2 (SARS-CoV-2) have been reported in the literature. However, the knowledge on Aspergillus co-infection among patients with coronavirus disease 2019 (COVID-19) is limited. COVID‐19‐associated pulmonary aspergillosis (CAPA) has been seen in critically ill COVID-19 patients with acute respiratory distress syndrome (ARDS), which has raised concerns about the worsening disease course of COVID-19 and increasing mortality. We describe a clinical case of CAPA infection and acute respiratory distress syndrome (ARDS) with a deathly outcome in a previously well, non-immunocompromised pregnant woman with intrauterine death of the fetus. Hence, we suggest that clinicians and pathologists keep alerting the possible occurrence of pulmonary aspergillosis in severe/critical COVID-19 patients, and aggressive investigations should be done to rule out the possibility of CAPA so that early treatment can be administrated.

## CASE REPORT

A 20-year-old woman presented to the emergency department with generalized tonic-clonic seizures. The patient was diagnosed with disseminated intravascular coagulation (DIC), intrauterine death of the foetus (IUD), multi-organ dysfunction syndrome (MODS) and septicaemia at the 18^th^ week of gestation. She was tested positive for coronavirus (COVID-19) by RT-PCR in a private nursing home. Her laboratory test showed hemoglobin 9.3 gm/dL (reference range [RR]; 12.0-15.0 gm/dL), total leukocyte count 22.8x10^9^/L (RR; 4.0-11.0 x10^9^/L), the platelet count was 108.9 x10^9^/L (RR; 150.0-400.0 x10^9^/L). Prothrombin time (test/control) was 24.4/11 sec, APTT 44.1/30, D-dimer 2880 ng/dL (RR; 200-500 ng/dL), fibrinogen 270 mg/dL (RR; 150-400 mg/dL), total bilirubin 4.6 mg/dL (RR; 0.3-1.2 mg/dL), direct bilirubin 2.28 mg/dL (RR;0.0-0.2 mg/dL), AST 120 U/L (RR; 0.0-35.0 U/L ), ALT 162 U/L (RR; 0.0-35.0 U/L), ALP 326.6 U/L (RR; 30.0-120.0 U/L ), GGT 16.7 U/L (RR; 0-38.0 U/L). urea 47.2 mg/dL (RR; 17.0-43.0 mg/dL), creatinine 2.81 mg/dL (RR; 0.55-1.02 mg/dL), sodium 138.1mmol/L (RR; 136-146 mmol/L) and potassium 3.96 mmol/L (RR; 3.5-5.1 mmol/L). Blood culture showed no growth after 48 hours of aerobic incubation in automated BACT/3D.

**Figure 1:**
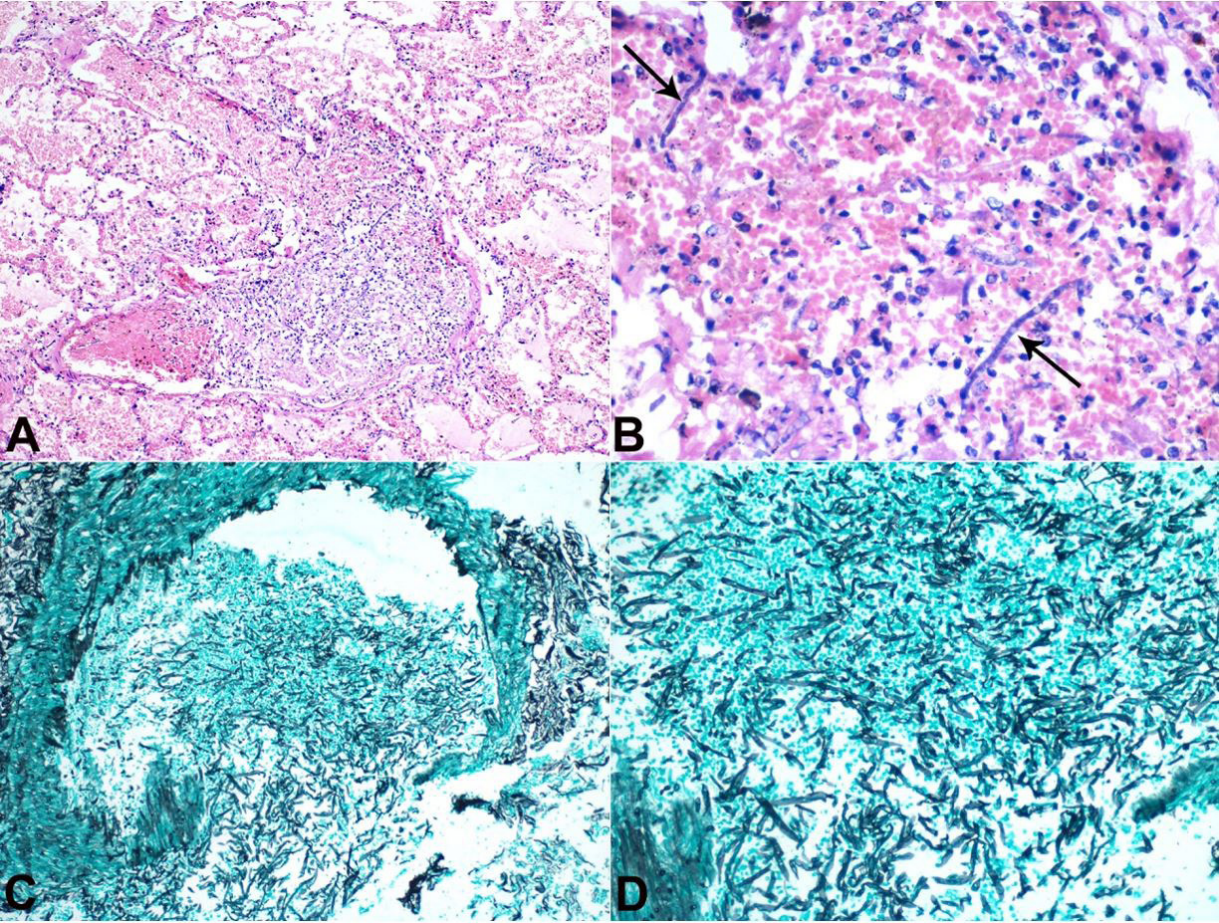
Photomicrograph of the lung. **A** – lung parenchyma with alveolar spaces filled by pink acellular proteinaceous fluid. Also, a large vessel containing mixed inflammatory cell infiltrate and areas of necrosis (H&E, 100X); **B** – numerous polymorphonuclear leucocytes, nuclear debris, red blood cells, and fungal hyphae with septations (arrows)(H&E 400X); **C** and **D** – Grocott-Gomori's Methenamine Silver (GMS) stain special stain demonstrating thick and thin-walled blood vessels with lumen filled with many fungal hyphae and disruption of the vessels wall (200X).

She was immediately managed symptomatically. However, the patient’s condition deteriorated despite all efforts, and she died on the second day of admission. With informed consent, the patient’s autopsy was performed under strict biosafety rules, and histopathological examination was performed (Figure 1).

**Figure 2:**
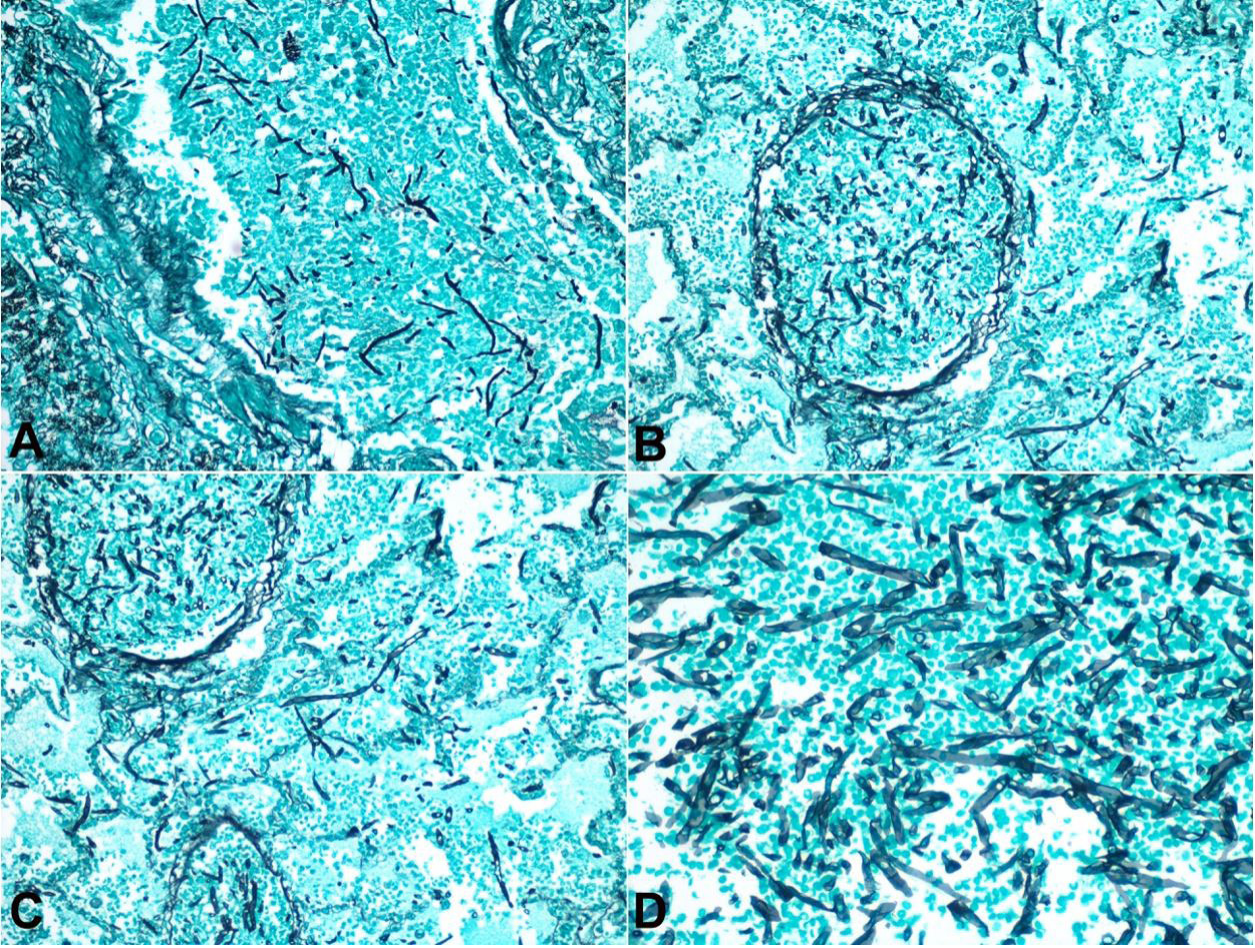
Photomicrograph of the lung. **A**, **B**, and **C** – demonstrating thick and thin-walled blood vessels with the lumen filled with many fungal hyphae and disruption of the vessels wall (Grocott-Gomori's Methenamine 200X); **D** – Higher magnification showing several fungal hyphae with acute angle branching and frequent septations morphologically consistent with aspergillosis (Grocott-Gomori's Methenamine 400X).

Histopathological examination from the lung revealed diffuse interstitial edema along with acute and chronic inflammation (Figure 1A, B), hematoxylin and eosin). Numerous septate fungal hyphae [red arrow] with acute-angled branching were found invading blood vessels (Figure 1 C, 2AC), Grocott-Gomori's Methenamine Silver), The septate hyphae morphology was consistent with Aspergillus species (Figure 1 D and 2D).

**Figure 3:**
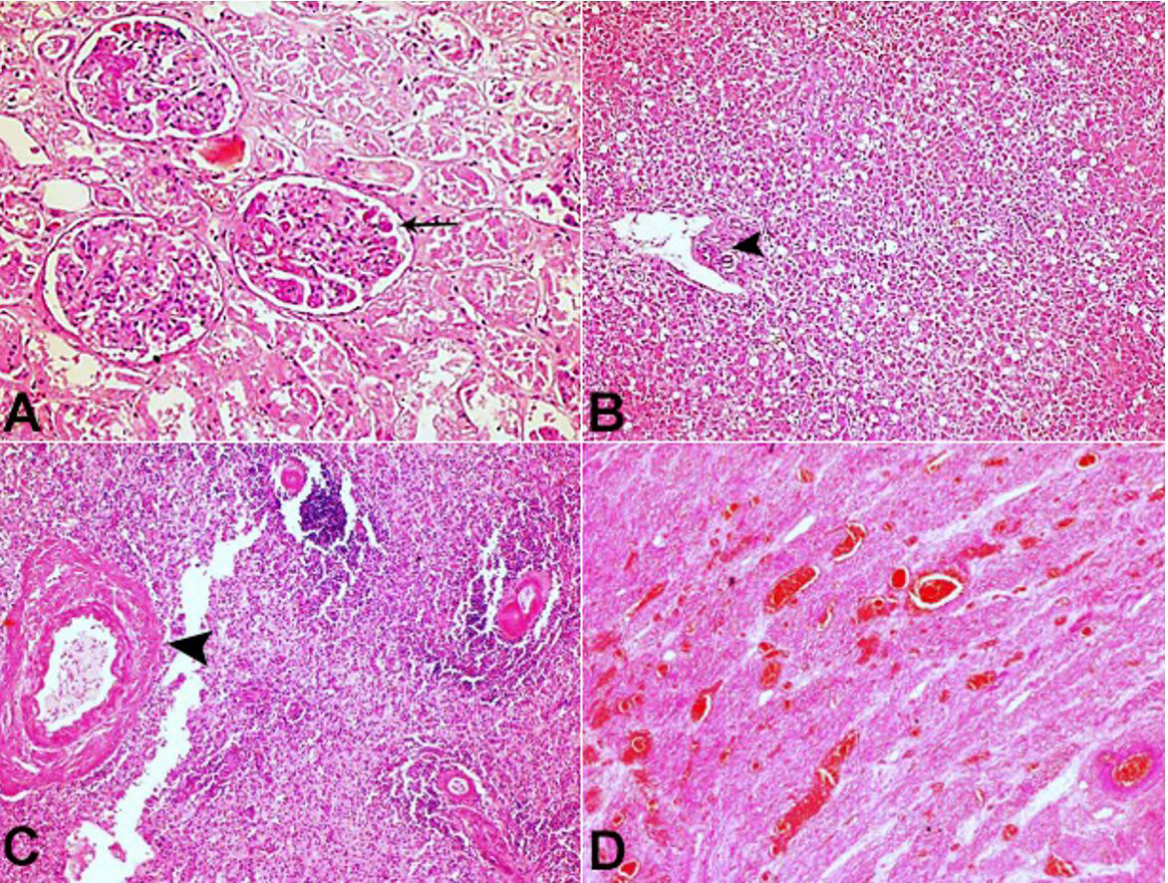
**A** – Glomeruli capillary filled with fibrin thrombi (arrow) along with acute tubular necrosis (ATN) (H&E x400); **B** – Necrosis of zone 1 to 3 along with portal track (arrowhead) from the liver (H&E x200); **C** – Section from spleen showing pan mural necrosis of the vessel wall (arrowhead) along with occasional viable lymphoid germinal center (H&E x400); **D** – The uterine section shows intramural thrombi in the vessel walls and necrosis and splaying of the smooth muscle fibers (H&E x200).

Histopathological examination from the other organs (Kidney, Liver, Spleen, and Uterus) showed fibrin thrombi in the vessels along with the variable degree of tissue necrosis. However, they did not reveal Invasive aspergillus Infection (Figure 3 AD).

## DISCUSSION

Following the recognition of coronavirus disease, 2019 (COVID-19) as a pandemic by the World Health Organisation in March 2020, COVID-19 associated-pulmonary aspergillosis (CAPA) reports are consistently being mentioned in the literature.^1^^-^ 3 Until recently, it was known that patients with acute respiratory distress syndrome due to viral infections such as influenza and SARS-CoV-2 were prone to secondary complications, especially in immunocompromised patients. Detection of aspergillosis in COVID-19 cases has been a new development wherein virus-induced severe immune dysfunction and damage to airway epithelium have been postulated to be the etiopathology for homing the fungi.^4^ In fact, concerns are being raised that the fungal superinfection could predispose patients with COVID-19 to a clinically worse disease contributing to the disease-associated fatality.^1^^,^ 3 We here report a case of CAPA in a young pregnant woman diagnosed with COVID-19 with no prior history of chronic immunosuppression.

Although the patient in our case had multiple contributing factors causing her mortality, it is possible that aspergillosis played a significant role in worsening the clinical scenario. The patient died despite active management with vasopressors, mechanical ventilation, and antimicrobials. A possible reason for this could be a lack of antifungal support, which would have hampered the disease progression. Furthermore, a history of IUD leading to DIC and subsequent MODS, in this case, masked the symptoms of pulmonary aspergillosis. This was compounded by normal chest X-ray findings. The possibility of nosocomial infection was excluded as the patient died the very next day of her hospitalization. Therefore, the present case emphasizes the role of autopsies for post-mortem surveillance in such a severe disease. Also, increasing availability of reports explaining the association of aspergillosis with COVID- 19 mandates screening for the fungi through non-invasive procedures such as a fungal culture or molecular studies to facilitate early detection of the fatal organism and prompt management of the disease with antifungal drugs, thereby avoiding a fatality^1^^,^ 2 ^,^ 5. Further research is necessary to evaluate the incidence of CAPA in SARS-CoV-2 infection and determine the population at risk, prophylaxis, and precise therapy.

## References

[1] Koehler P, Bassetti M, Chakrabarti A (2020). Defining and managing COVID-19-associated pulmonary aspergillosis: the 2020 ECMM/ISHAM consensus criteria for research and clinical guidance. Lancet Infect Dis.

[2] Nasir N, Farooqi J, Mahmood SF, Jabeen K (2020). COVID-19-associated pulmonary aspergillosis (CAPA) in patients admitted with severe COVID-19 pneumonia: an observational study from Pakistan. Mycoses.

[3] Arkel ALE, Rijpstra TA, Belderbos HNA, Wijngaarden P, Verweij PE, Bentvelsen RG (2020). COVID-19-associated Pulmonary Aspergillosis. Am J Respir Crit Care Med.

[4] Qin C, Zhou L, Hu Z (2020). Dysregulation of immune response in patients with Coronavirus 2019 (COVID-19) in Wuhan, China. Clin Infect Dis.

[5] Blot SI, Taccone FS, Abeele AM (2012). A clinical algorithm to diagnose invasive pulmonary aspergillosis in critically ill patients. Am J Respir Crit Care Med.

